# iTRAQ-Based Proteomic Analysis Reveals the Role of the Biological Control Agent, *Sinorhizobium fredii* Strain Sneb183, in Enhancing Soybean Resistance Against the Soybean Cyst Nematode

**DOI:** 10.3389/fpls.2020.597819

**Published:** 2020-12-11

**Authors:** Yuanyuan Wang, Ruowei Yang, Yaxing Feng, Aatika Sikandar, Xiaofeng Zhu, Haiyan Fan, Xiaoyu Liu, Lijie Chen, Yuxi Duan

**Affiliations:** ^1^College of Bioscience and Biotechnology, Shenyang Agricultural University, Shenyang, China; ^2^Nematology Institute of Northern China, Shenyang Agricultural University, Shenyang, China; ^3^College of Sciences, Shenyang Agricultural University, Shenyang, China

**Keywords:** soybean cyst nematode, Sinorhizobium fredii, induced resistance, biological control, isoflavonoid biosynthesis

## Abstract

The soybean cyst nematode (SCN), *Heterodera glycines* Ichinohe, poses a serious threat to soybean production worldwide. Biological control agents have become eco-friendly candidates to control pathogens. Our previous study indicated that the biocontrol agent, *Sinorhizobium fredii* strain Sneb183, may induce soybean resistance to SCN. To study the mechanisms underlying induced disease resistance in the plant by Sneb183, an iTRAQ (isobaric tag for relative and absolute quantitation)-based proteomics approach was used to identify proteomic changes in SCN-infected soybean roots derived from seeds coated with the Sneb183 fermentation broth or water. Among a total of 456 identified differentially expressed proteins, 212 and 244 proteins were upregulated and downregulated, respectively, in Sneb183 treated samples in comparison to control samples. Some identified differentially expressed proteins are likely to be involved in the biosynthesis of phenylpropanoid, flavone, flavanol, and isoflavonoid and have a role in disease resistance and adaptation to environmental stresses. We used quantitative real-time PCR (qRT-PCR) to analyze key genes, including *GmPAL* (phenylalanine ammonia-lyase), *GmCHR* (chalcone reductase), *GmCHS* (chalcone synthase), and *GmIFS* (isoflavone synthase), that are involved in isoflavonoid biosynthesis in Sneb183-treated and control samples. The results showed that these targeted genes have higher expression levels in Sneb183-treated than in control samples. High performance liquid chromatography (HPLC) analysis further showed that the contents of daidzein in Sneb183-treated samples were 7.24 times higher than those in control samples. These results suggested that the *Sinorhizobium fredii* strain Sneb183 may have a role in inducing isoflavonoid biosynthesis, thereby resulting in enhanced resistance to SCN infection in soybean.

## Introduction

Soybean [*Glycine max* (L.) Merr] is one of the most important crops for human consumption and livestock feed and has diverse applications for industrial products. The most economically damaging plant-parasitic nematode (PPN) is *Heterodera glycines* (Williamson and Gleason, 2003). In China, yield losses due to *H. glycines* have been estimated at more than 120 million USD and approximately 1.5 billion USD worldwide (Wrather et al., 2001; Moens et al., 2008; Hosseini and Matthews, 2014). Chemical nematicides, as the main management technique, are used to reduce nematode incidence. However, they are being withdrawn from use due to their toxicity to humans and their impact on the environment. Thus, there is an increased demand for eco-friendly products to manage *H. glycines*.

Rhizobia are a diverse group of nodule-forming bacteria known for inhabiting the soil and establishing functional symbiotic associations with legume plants. The symbioses between legumes and different genera of rhizobia, including *Rhizobium*, *Bradyrhizobium*, and *Mesorhizobium*, are a cheaper source of N and an effective agronomic practice that ensure an adequate supply of N compared to the N fertilizer (Subramanian et al., 2004). They not only play a major role in biological nitrogen fixation but also improve plant growth and reduce disease incidence in various crops. Some rhizobia strains have also been reported as biocontrol agents (BCAs) for the control of diseases caused by parasitic nematodes through direct and/or indirect mechanisms. Siddiqui et al. (2007) studied the biocontrol of *Meloidogyne javanica* in lentils inoculated with the selected strain of *Rhizobium* as single and co-inoculations, which significantly reduced galls per root system and population of nematodes per kg soil, in addition to causing a greater increase in the plant growth in the absence of *M. javanica*. Ashoub and Amara (2010) demonstrated the ability of a *Rhizobium* strain isolated from the broad bean (*Vicia faba*) to cause 100% juvenile mortality of *Meloidogyne incognita in vitro* for 72 h. *Rhizobium* spp. efficiently reduced the *M. javanica* infection on eggplant (*Solanum melongena*; Mohamedova et al., 2016). Even individual cellular components of the *Rhizobium* had been shown to induce ISR viz. lipopolysaccharides (LPSs), flagella, cyclic lipopeptides, homoserine lactones, acetoin, and butanediol (Lugtenberg and Kamilova, 2009).

Isoflavonoids are specialized metabolites of dual importance for plant-environment interactions. They act as signaling molecules in symbiotic nitrogen fixation by Rhizobia (Phillips and Kapulnik, 1995; Ferguson and Mathesius, 2003; Subramanian et al., 2006) and as phytoalexins with antimicrobial properties (Lozovaya et al., 2004; Lygin et al., 2013). Isoflavonoids are synthesized through a legume-specific branch of the phenylpropanoid pathway, which is regulated by the coordinated expression of several structural genes. *PAL*, *CHS*, *C4H*, *CHR*, and *IFS*, considered as markers for the isoflavonoid biosynthetic pathway, exist intrinsically in the cell but are sometimes activated under certain conditions (Dao et al., 2011; Anguraj Vadivel et al., 2019), which are induced and regulated by internal and external factors, and they regulate isoflavonoid biosynthesis at both the transcription and post-transcriptional levels (Nakatsuka et al., 2003; Arfaoui et al., 2016). Defense-induced expression of IFS and several other genes involved in isoflavonoid metabolism in alfalfa leaves was previously reported (He and Dixon, 2000). The expression pattern of IFS in soybean is consistent with the physiological roles of isoflavonoids as defense compounds against pathogens and signal molecules to symbiotic bacteria, which are related to the regulation of the expression of SA (salicylic acid) and JA (jasmonic acid) hormones in soybean (Subramanian et al., 2004). The isoflavonoids that accumulate at PPN feeding sites may affect nematode fertility and fecundity by limiting egg production or skewing the ratio of males to females, as more females are formed under abundant nutrition and vice versa (Volpiano et al., 2019), while the ratio of males to females is an important index because it determines the population size and the extent of damage (Triantaphyllou, 1973). Our previous study indicated that the BCA, *Rhizobium* strain Sneb183, originally isolated from the rhizosphere of pines in Liaoning, China, and was identified as *Sinorhizobium fredii* (Zhao et al., 2009), triggered an induced systemic resistance (ISR) to soybean cyst nematode (SCN) infection in soybean by split-root experiments (Tian et al., 2014). ISR induces the host physiological and metabolic responses, leading to the synthesis and accumulation of additional defensive chemicals in plants, which interfere with the invasion of plant pathogens.

This study was conducted to evaluate the molecular mechanisms of ISR by *S. fredii* strain Sneb183. The differentially expressed proteins (DEPs) involved in disease resistance and adaptation to environmental stresses in Sneb183 pre-treated samples were compared with those in control samples by iTRAQ (isobaric tag for relative and absolute quantitation) technology. The quantitative real-time PCR (qRT-PCR) and high performance liquid chromatography (HPLC) analyses were also conducted to evaluate the efficacy of Sneb183, which regulates the expression of isoflavonoid biosynthesis-related genes during SCN infection. The results of this analysis will serve as the theoretical framework for the production of marketable and useful BACs.

## Materials and Methods

### Isolation of Cysts and J2 of SCN

Surface soil samples (0–2 cm depth) were collected from the rhizosphere of soybean (*G. max* cv. Liaodou 15, an SCN-susceptible cultivar). Cysts were collected following elutriation and hand-picking under a stereomicroscope and surface-sterilized by immersion in a 0.1% HgCl_2_ solution for 1 min, followed by rinsing three times with the sterile distilled water. They were then placed in the Baermann funnel at 25°C. J2s were collected from the bottom of the Baermann funnel every 2 or 3 days (Liu, 1995).

### Preparation of the *Sinorhizobium fredii* Sneb183 Fermentation Broth

The *S. fredii* strain Sneb183, preserved at −80°C, was suspended in the sterilized water and adjusted to 1.0 × 10^8^ CFU/ml with a hemocytometer under a microscope, followed by the addition of 1.0 ml of this suspension to 50 ml of the sterilized TY liquid medium (Duelli and Noel, 1997). The Sneb183 fermentation broth was maintained at 28°C and 150 rpm for 168 h for the subsequent use.

### Plant Materials and Treatments

The soybean cultivars Liaodou 15, which are susceptible to SCN, were used. The seeds were surface-sterilized with 0.5% NaClO for 10 min, washed several times with sterile distilled water, and air-dried. The sterilized seeds were equally coated with fermentation broth of Sneb183 with a ratio of 70:1 (g/ml), and distilled water was used as control. For seed coating, fermentation of Sneb183 was added and properly mixed for uniform coating on all seed and left for complete drying. They were then germinated on filter paper in distilled water in Petri dishes and incubated at 28 ± l°C for a week. The seeds were sown in 18-cm plastic pots containing a sterile soil mixture (topsoil:sand:vermiculite; 3:2:1). The seedlings were separated into four treatments until the Vc stage. The treatments were (1) seed coated with the Sneb183 fermentation broth (Sneb183), (2) inoculated with 2,000 J2s of SCN (J2), (3) seed coated with the Sneb183 fermentation broth and inoculated with 2,000 J2s of SCN (Sneb183 + J2), and (4) seed coated with the distilled water (CK). After 7 days of inoculation of J2s, the soybean seedlings from the treatments (2) and (3) were transferred to new plastic containers filled with the sterile soil mixture and irrigated with 1/4 Hoagland nutrient solution in a growth chamber under normal conditions (25/20°C day/night temperature, the relative humidity of 60–80%, and 16 h light/day with the intensity of 160 μmol photons m^−2^ s^−1^). At 3, 7, 14, 21, and 28 days after the inoculation, the treated soybean root samples were collected at proteomic and transcript levels. Three independent sets of four treatment samples were collected, and each replicate represented a pooled sample of three individual plants.

### Protein Extraction and Quantification

At 7 days post-inoculation (dpi), the proteomics analysis was performed using root samples and total protein from three biological replicates was prepared from four treatments. For each sample group, the total protein was extracted from root samples using trichloroacetic acid (TCA) and acetone (Méchin et al., 2007) with the following modifications. Briefly, about 0.5 g of soybean root samples was pulverized to a fine powder in liquid nitrogen using a mortar and pestle. The root proteins were suspended in pre-cooled 10% (w/v) TCA in acetone and incubated at −20°C for 2 h. The mixture was then centrifuged at 20,000 *g* for 30 min at 4°C. The protein pellets were thoroughly washed twice with the pre-cooled acetone and dried in a SpeedVac. The dried pellets were resuspended with the lysis buffer containing 8 M urea, 30 mM HEPES, 1 mM PMSF, 2 mM EDTA, and 10 mM dithiothreitol (DTT), with the pH of 8.8, sonicated for 5 min (pulse on 2 s/off 3 s power, 180 W), and centrifuged at 20,000 *g* for 30 min. DTT was added to 10 mM in a 56°C water bath for 1 h and IAM was added to 55 mM, and they were placed in the dark for 1 h. The volume of four times greater than the volume of the sample solution was added to the pre-cooled acetone at −20°C for 3 h. The precipitate was obtained by centrifugation at 20,000 *g* at 4°C for 30 min. Around 500 μl buffer was added, sonicated for 3 min, and centrifuged at 20,000 *g* at 4°C for 30 min. The supernatants were collected and stored at −80°C for further use. Protein concentrations were determined using the Bradford assay (Bio-Rad) using BSA as the standard.

### Protein Digestion and iTRAQ Labeling

Around 100 μg of protein sample was digested with trypsin at 20:1 (w/w) at 37°C for 36 h. The digest was lyophilized, and the peptide was reconstituted in 30 μl of TEAB per tube (water:TEAB = 1:1, containing 0.1% SDS). After digestion, 1 μl peptide segments were labeled using the iTRAQ Reagents 8 plex kit, according to the manufacturer’s instructions (AB Sciex Inc., United States). The Sneb183, J2, Sneb183 + J2, and CK were labeled as iTRAQ tags 113, 115, 117, and 119, respectively. Three sets of iTRAQ samples were used for the three biological replicates. After labeling, samples were combined and lyophilized. The peptide mixture was dissolved in strong cation exchange (SCX) solvent A containing 2% (v/v) acetonitrile, 20 mM ammonium formate, and 25% ammonium hydroxide with the pH of 10.0, and then fractionated on the Agilent HPLC system 1100 with Phenomenex Luna C18 column (250 × 4.6 mm, 5 μm, 100 Å). Peptides were eluted with a flow rate of 200 μl min^−1^ and a gradient of 0–12% buffer B containing 85% (v/v) acetonitrile, 20 mM ammonium formate, and 25% ammonium hydroxide with the pH of 10.0 for 1 min, 12–56% buffer B for 35 min, and 56–100% buffer B for 5 min, followed by ramping up to 100% solvent B for 5 min. The absorbance was measured at 280 nm, and a total of 16 fractions were collected.

### Protein Identification by Mass Spectrometry

Each SCX fraction was lyophilized, dissolved in 1 ml MilliQ water, and loaded onto a C18 nanoflow column (75 mm internal diameter, 5 μm, 300 Å). Peptides from iTRAQ samples were separated using a gradient of 65 min, ranging from 95% solvent A containing water and 0.1% formic acid/5% solvent B containing acetonitrile and 0.1% formic acid for 10 min, followed by 5–30% solvent B for 30 min, 30–60% solvent B for 5 min, and 60–80% solvent B for 3 min, holding 80% solvent B for 7 min, 80–5% solvent B for 3 min, and 5% solvent B for 7 min.

MS/MS analysis was carried out on a Q Exactive mass spectrometer (Thermo Scientific, Bremen, Germany). Briefly, a full MS survey scan was performed with a mass range of 350–2,000 m/z and a resolution of *R* = 17,500. HCD fragmentation was used for MS/MS analysis, and the 10 most intense signals in the survey scan were fragmented.

### Analysis and Proteomic Interpretation of Data

The raw files were converted to Mascot generic format (.mgf) files using Proteome Discoverer 1.4 (Thermo Fisher Scientific, Waltham, MA, United States) with default settings for deep proteome analysis. The following parameters were used for searching: the minimum and maximum precursor mass of 350 and 6,000 Da, respectively; minimum S/N ratio of 1.5; enzyme:trypsin; maximum missed cleavages of 1; FDR ≤ 0.01; and mass tolerance of 15 ppm for precursor ions. Mascot (Matrix Science, London, V2.3.0) was used for deep proteome and protein quantitation analyses with .mgf files as input.

In order to be identified as substantially differentially expressed, a protein should be quantified with at least three peptides in each experimental replicate, with a value of *p* smaller than 0.05 and a fold change of greater than 1.2. The searches were performed against the Swiss-Prot GreenPlants database (Release 2012_05, Number of sequences: 538585).

The functional annotation of proteins obtained was determined by Blast2GO^1^ (Bioinformatics Department, CIPF, Valencia, Spain), and WEGO^2^ was used to enrich the GO annotation results. To enrich pathways for DEP, KEGG,^3^ was used to estimate the metabolic pathways involved in the defense response of soybean to the infection to optimize pathways for DEP.

### RNA Extraction and Quantitative Real-Time PCR

The key enzyme genes involved in the isoflavonoid biosynthesis in different treatments were validated by qRT-PCR to verify the quality of the iTRAQ-based proteomic analysis. Total RNA was extracted from soybean roots from four treatments separately by the RNA pure Plant Kit (CWBIO, Beijing, China), according to the manufacturer’s instructions. The RNA quality was tested by 1% agarose gel electrophoresis, and concentrations and purity were determined using a NanoVue plus spectrophotometer (GE, MA, United States). cDNA was reverse-transcribed from 1 μg of total RNA using the RT Reagent Kit (Takara). qRT-PCR was performed using the CFX Connect Real-Time PCR Detection System (Bio-Rad, CA, United States). Gene-specific primers (GSPs) used for *PAL*, *CHS*, *CHR*, and *IFS* were described in Supplementary Table S1. The soybean *actin 11* gene was used as an endogenous control for normalization. The 20-μl reaction mixture contained 10 μl 2 × TB Green premix (Takara), 1 μl cDNA template (1:5 dilution), 0.4 μl 50 × ROX Reference DyeII (Takara), 7 μl SDW, and 0.8 μl of each forward and reverse primers for selected genes. The thermal cycling conditions of qRT-PCR for all reactions were 95°C for 1 min and 50 s, followed by 40 cycles of 95°C for 10 s, 55°C for 33 s, and 68°C for 30 s. There were three biological replicates and three technical replicates per time-point for each treatment. The qRT-PCR data were analyzed using the 2^–*Δ*ΔCt^ relative quantification method (Livak and Schmittgen, 2001).

### Measurement of Genistein and Daidzein Contents in Soybean Roots

About 0.5 g of fresh hairy roots of soybean from four treatments were accurately weighed and extracted with 50 ml of 70% ethanol at 60°C for 2 h. The homogenates were centrifuged at 10,000 *g* for 10 min, evaporated, and resuspended in 80% methanol. Genistein and daidzein contents were determined using the A262 HPLC–UV System (Agilent 1100 series, DAD, Agilent Technologies, Palo Alto, CA, United States) on a Platisil ODS C18 column (3 μm, 250 × 4.6 mm, Dikmatech). The digests were then separated by a linear gradient ranging from 87% solvent A (0.1% acetic acid)/13% solvent B (0.1% acetonitrile acetate) to 50% solvent A/50% solvent B in 87 min at a flow rate of 1.2 ml min^−1^. The temperature of the column and the volume of injection were 40°C and 10 μl, respectively. All measurement experiments were performed at least in triplicate.

### Statistical Analysis

Statistical analysis was performed using a two-way ANOVA using Graphpad Prism v.8.0. Significant differences between group means were determined by the Tukey’s multiple comparisons test at *p* < 0.05.

## Results

### Protein Identification by iTRAQ Analysis

Changes in the proteome of soybean roots from four treatments were analyzed using iTRAQ-LC/MS-MS technology. Data from three biological replicates were analyzed, and proteins were detected by querying data using a soybean protein database. Among a total of 443,104 spectra, 25,406 could be matched to the database, resulting in a total of 4,193 peptides assembled into 1,366 protein groups with high confidence (confidence level > 95%).

**Figure 1:**
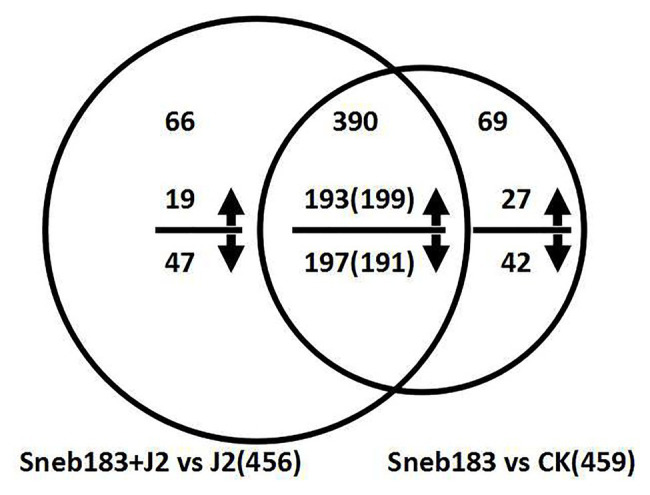
Venn diagram of differentially expressed proteins responsive in seed-coated with Sneb183 fermentation broth then inoculated J2 of SCN (Sneb183 + J2) vs. inoculated J2 of SCN (J2) and seed-coated with Sneb183 (Sneb183) vs. CK. The number above or below the horizontal line in each portion indicated the number of upregulated or downregulated proteins. The over lapping regions indicated the number of common proteins. Among the 390 common DEPs, 193 were upregulated and 197 were downregulated in Sneb183 + J2 vs. J2; and 199 were upregulated and 191 were downregulated Sneb183 vs. CK.

According to the quantitative data of the Swiss-Prot GreenPlants library protein, DEPs were selected with more than 1.2-fold differences, the expression levels at *p* < 0.05, and a false discovery rate of less than 1%. Based on these rules, 459 DEPs were identified in soybean roots (Figure 1, Supplementary Table S2), of which 226 (49.2%) showed an increase and 233 (50.8%) showed a decrease in abundance between Sneb183 vs. CK treatment. At the same time, 456 DEPs in soybean roots, including the increased numbers of 212 (47.6%), and decreased numbers of 244 (52.4%) were identified between Sneb183 + J2 vs. J2 treatment (Figure 1, Supplementary Table S3). The further function annotations of 456 identified DEPs demonstrated that these proteins were involved in a variety of metabolisms, such as energy, carbohydrate, and amino acid. Besides, some DEPs were also enriched in pathways such as energy metabolism, systemic resistance, and adaptation to environmental stresses, including calcium signaling pathway, isoflavonoid biosynthesis, pentose phosphate pathway, glutathione metabolism, etc. The findings revealed the responses to Sneb183 inoculation at the level of protein in the soybean core. They could provide valuable tools for more research on the *S. fredii* strain Sneb183 ISR’s molecular mechanism against the soybean cyst nematode. In the meanwhile, the identification of proteins from other treatments was shared (Supplementary Tables S4, S5).

### Protein Annotation and Enrichment

**Figure 2:**
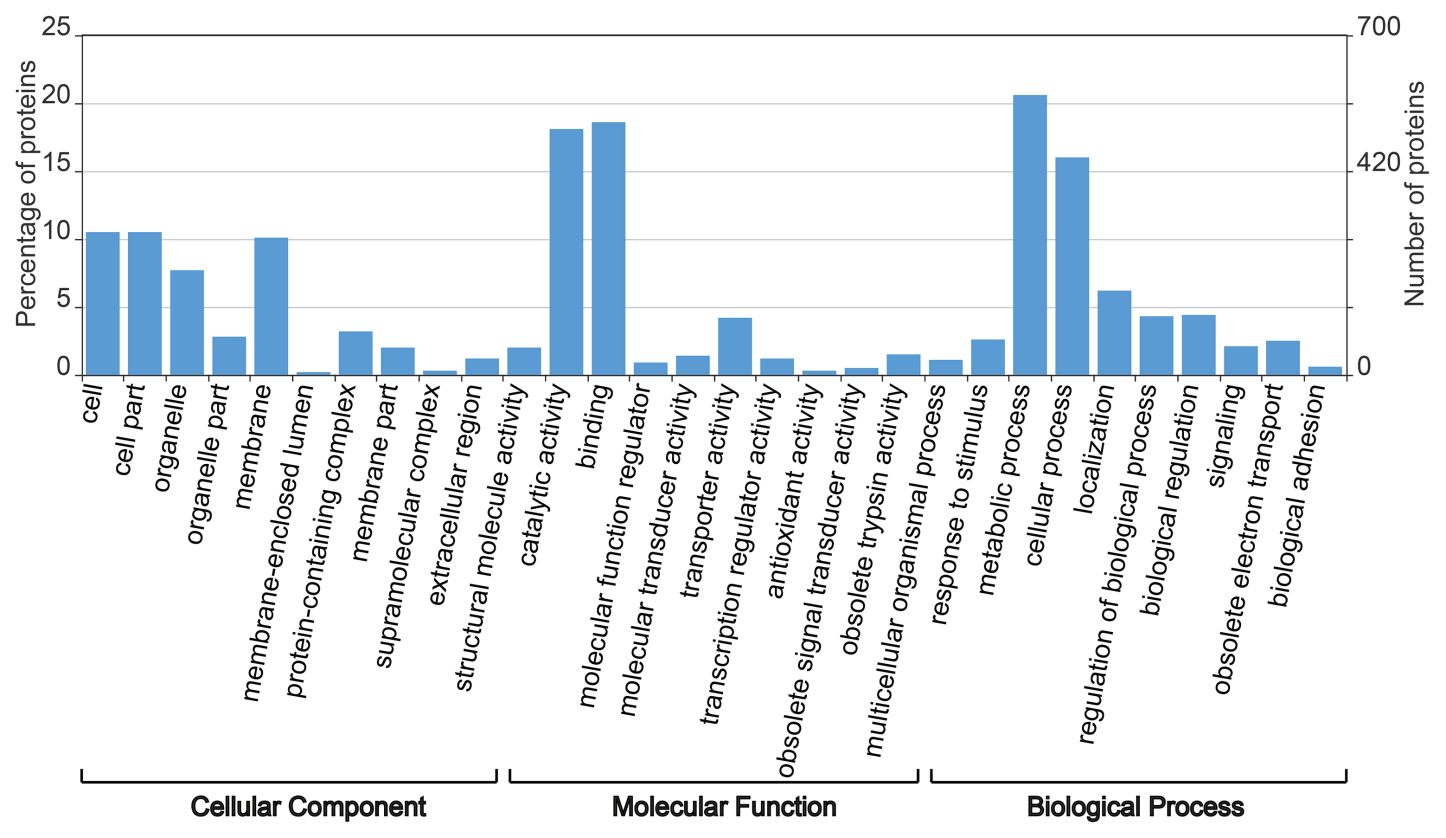
Gene ontology annotations for all differentially expressed proteins in seed-coated with Sneb183 fermentation broth then inoculated J2 of SCN (Sneb183 + J2) vs. inoculated J2 of SCN (J2).

According to the BLAST database, Gene Ontology annotation was performed based on all DEPs of Sneb183 + J2 vs. J2. A total of 2,832 GO terms, including 979 biological words, 568 cell words, and 1,285 feature terms were annotated as a result of GO annotation of the protein-coding genes. The results of the annotation showed that 578, 520, 507, 448, and 294 genes were enriched in the metabolic process, binding, catalytic activity, cell process, and cell part, respectively (Figure 2). This result suggests that the *S. fredii* strain Sneb183 may improve the resistance to *H. glycines* by affecting the metabolic process of soybean.

**Figure 3:**
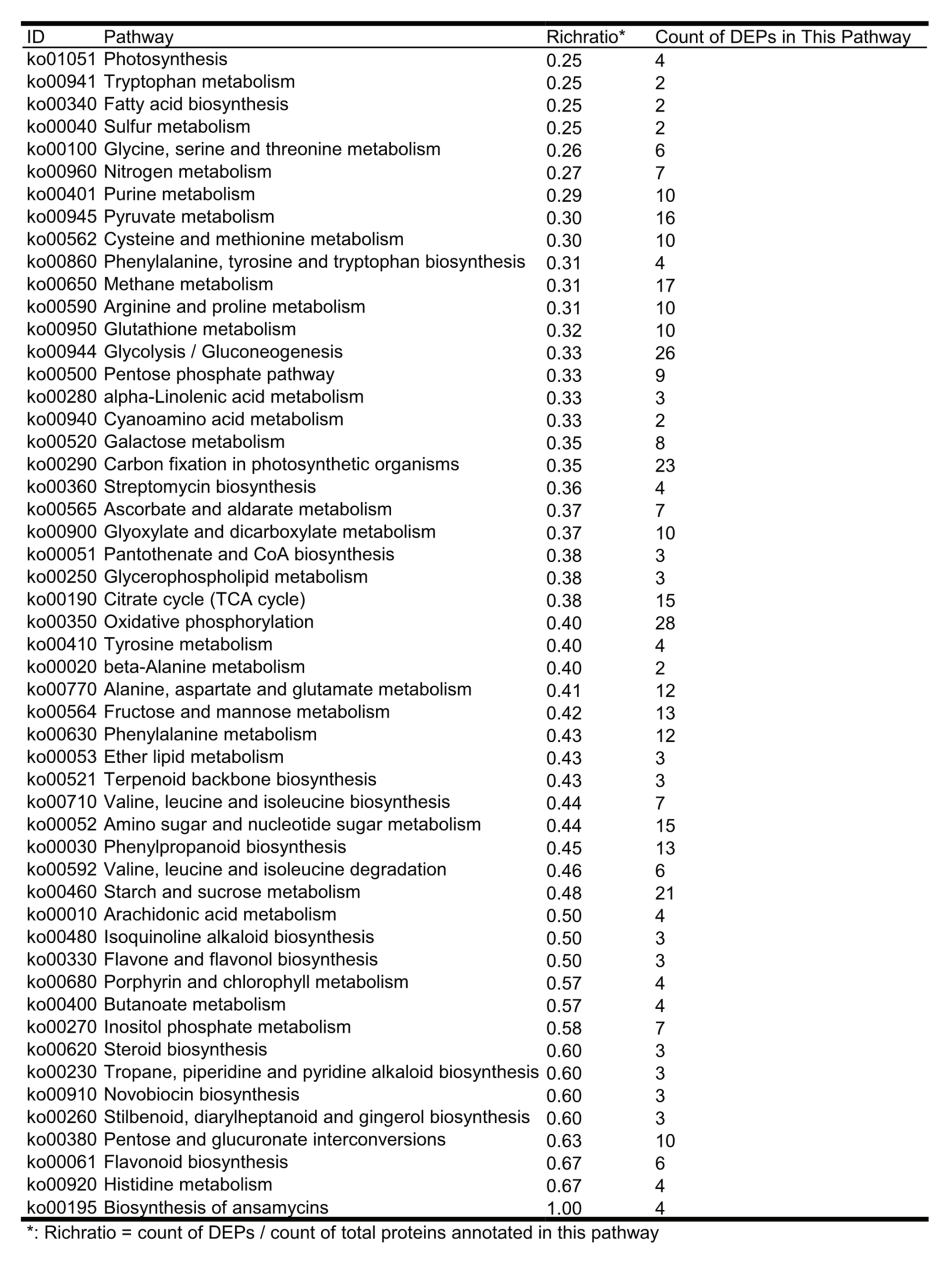
The count of DEPs in seed-coated with Sneb183 fermentation broth then inoculated J2 of SCN (Sneb183 + J2) vs. inoculated J2 of SCN (J2) in each metabolic pathways.

**Figure 4:**
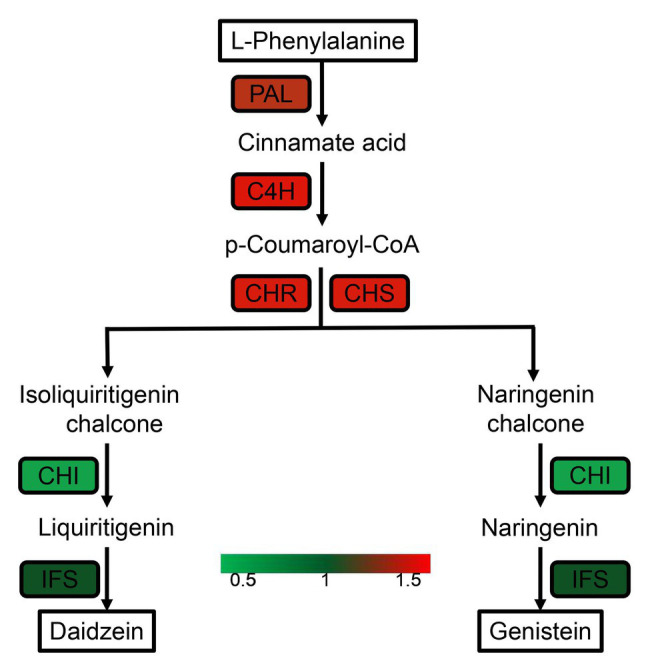
DEPs in seed-coated with Sneb183 fermentation broth then inoculated J2 of SCN (Sneb183 + J2) vs. inoculated J2 of SCN (J2) associated with isoflavonoid biosynthesis pathway. Different colors reflect the changes in protein abundance based on the iTRAQ data.

The results of the KEGG study showed that 337 out of 456 DEPs involved in more than 118 metabolic pathways were identified; some proteins are involved in multiple metabolic pathways at the same time, suggesting that these proteins have multiple biological roles. The identified DEPs are involved in some of the metabolic pathways related to plant disease resistance and adaptation to stressful environments, and the enrichment occurs in pathways (Figure 3), such as biosynthesis of phenylalanine, tyrosine, and tryptophan (map00400), phenylpropanoid biosynthesis (map00940), flavonoid biosynthesis (map00941), and flavone and flavanol biosynthesis (map00944). We further summarized the list of DEPs involved in flavonoids and isoflavonoid pathways associated with plant resistance (Supplementary Table S6). Our results also indicated that four DEPs upregulated were related to the isoflavonoid metabolism pathway (Figure 4). However, further studies are required to investigate the metabolic pathways associated with other DEPs.

### Regulation of Some Proteins Involved in Isoflavonoid Biosynthesis at the mRNA Level

**Figure 5:**
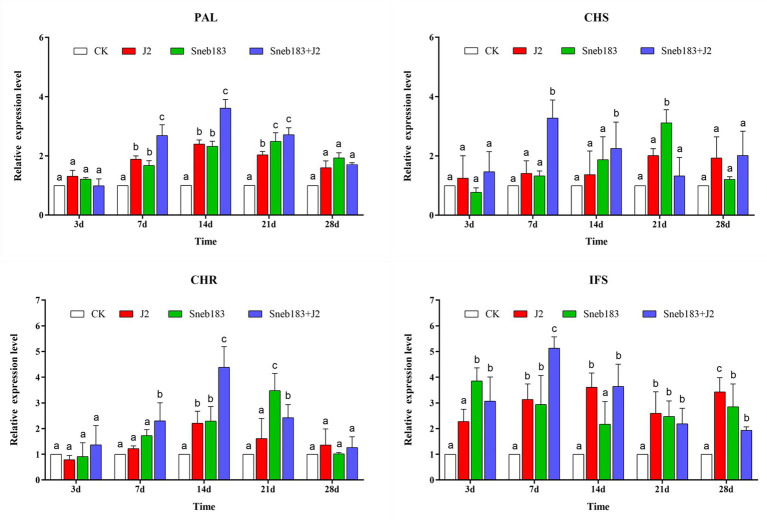
Quantitative RT-PCR analysis of key genes involved in the isoflavonoids synthesis pathway in the root. Gene expression levels were analyzed from four treatments: CK, J2, Sneb183, and Sneb183 + J2 at 3, 7, 14, 21, and 28 days after *H. glycines* infection. Relative transcript levels were compared with those of CK for each time point. Error bars represent standard error of the mean values of three technical replicates from three biological replicates. The differences in expression levels were tested by two-way ANOVA using Graphpad Prism v.8.0. According to Tukey’s multiple comparisons test at *p* < 0.05. For each gene and time point, bars with different letters indicate statistically significant differences between the treatments.

The *S. fredii* Sneb183 induced resistance against SCN in soybean, and some DEPs involved in pathways for biosynthesis of flavone and isoflavonoid were enriched. Accordingly, in our study, qRT-PCR was used to further analyze and verify the key genes, including *GmPAL*, *GmCHS*, *GmCHR*, and *GmIFS* in the isoflavonoid biosynthesis pathway (Figure 5).

After 7 dpi, a significantly and consistently enhanced transcription of *GmPAL* was induced in the treatments J2/Sneb183/Sneb183 + J2 compared to CK. The transcript level of *GmPAL* was not different at 3 dpi when the J2 feeding sites were established. At 7 and 14 dpi, during J2 development in roots, the expressions of *GmPAL* in Sneb183 + J2 increased by 2.7-fold and 3.6-fold, respectively, which were 1.42-fold and 1.51-fold greater than those in the J2 treatment at the same time point. As J2s developed to the adult stage, the *GmPAL* transcription was enhanced in the treatments J2, Sneb183, and Sneb183 + J2 compared to CK, with no differences between the treatments. The transcript levels of *GmCHS* and *GmCHR* showed a similar trend in four treatments. No difference was observed at 3 dpi; however, at 7 and 14 dpi, the expression of *GmCHS* in Sneb183 + J2 was significantly higher than that in CK, J2, and Sneb183 during the development of nematode in roots. In Sneb183 + J2, which was 5.89-fold higher than that in J2, the highest expression level of *GmCHS* occurred at 7 dpi. In Sneb183 + J2, *GmCHR* had the largest 2.18-fold greater expression level than in J2 at 14 dpi. However, at 28 dpi, *GmCHS* and *GmCHR* expression levels decreased slightly in both treatments.

The expression pattern of *GmIFS* was as follows: in each treatment, in the early stage (at 3 and 7 dpi) of the nematode development, the expression level was higher than that in the later stage (14, 21, and 28 dpi). On the 7th day, the expression level in Sneb183 + J2 was the highest, 1.56 times greater than in J2, which was significantly different in other treatments.

### Measurement of Genistein and Daidzein Contents in Soybean Roots

**Figure 6:**
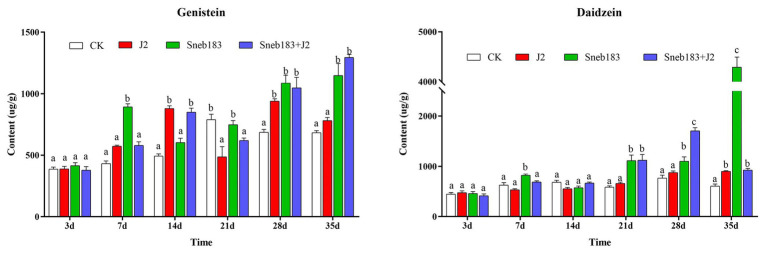
Contents of genistein and daidzein in soybean seedling at different times intervals. The error bars illustrated the mean contents of genistein or daidzein ± standard deviation (*n* = 3). The same letters on bars are significantly similar, according to Tukey’s multiple comparisons test (*p* < 0.05).

The observed influence of seed coating with Sneb183 on genes encoding key enzymes in the isoflavonoid biosynthesis pathway raised the question of whether such modifications would contribute to a substantial change in isoflavonoid concentrations. Therefore, the contents of two isoflavonoids (genistein and daidzein) in soybean roots, treated with Sneb183 from four treatments, were detected by HPLC. The accumulation of genistein and daidzein in response to different treatments is shown in Figure 6.

Genistein contents in soybean roots increased gradually from 7 days in all treatments. The seed coating with the Sneb183 fermentation broth resulted in a significant increase in genistein contents as compared to the other treatments at 7 days. Longitudinal comparison of the genistein content in each treatment, we found it reached the peak at 21 days in the CK treatment, while in treatments of J2 and Sneb183, the speed of genistein accumulation were accelerated and reached the peak at 14 and 7 days, respectively. Regarding daidzein content, it is higher than that of genistein in each treatment during the same period. Similarly to genistein, there was a significant difference in the content of daidzein compared to the other treatment at 7 days. There was a steady increase in daidzein content until 21 days in Sneb183 and Sneb183 + J2 treatments, followed by a dramatic increase. Daidzein content in all coating treatments with or without nematode inoculation significantly increased as compared to CK and J2 since 21 days. At 35 days, the contents of daidzein in Sneb183 treatment were 7.24 times higher than those in roots derived from control samples.

## Discussion

It is well-known that soybean [*G. max* (L.) err.] is economically the most important bean in the world (Mendoza-Suárez et al., 2020). SCN (*H. glycines* Ichinohe) is the most serious pest in soybean production, and the complete elimination of *H. glycines* from the soil is difficult due to its polyphagous nature. Plant growth-promoting bacteria (PGPB), colonize the rhizosphere of many plant species, are the most potential BCAs of plant-parasitic nematode. Numerous study indicated seed coating with PGPB, such as *Rhizobium etli*, *Bacillus firmus*, *B. subtilis*, *B. simplex*, *Klebsiella pneumoniae* (Hallmann et al., 2001; Siddiqui and Shaukat, 2003; Terefe et al., 2009; Kang et al., 2018; Liu et al., 2018) elicited systemic resistance against nematode disease. Previously studies verified that *S. fredii* Sneb183 ISR in the split-root system by seed coating and significantly reduce cysts on roots after seed coating with the Sneb183 fermentation. Admittedly, seed coating is a cost-effective and more efficient agricultural processing operation with strong operability, without altering their genome sequences, these plants obtain extensive resistance, respond more rapidly and strongly to various stresses (Mathre et al., 1999; Pathak et al., 2016; Pedrini et al., 2017).

The present study involved the comparative analysis of soybean roots by iTRAQ to investigate the plant resistance to SCN induced by the *S. fredii* strain Sneb183. Isoflavonoid, plant defense enzymes, phenolics or other phytoalexins, which are induced and accumulated in the plant in response to rhizobia, have been reported to protect plants from phytopathogens (Dakora, 2003). Flavones and isoflavonoides have usually been used as defense compounds against many plant-parasitic nematodes (Chin et al., 2018). Carpentieri-Pípolo et al. (2005) demonstrated that soybean isoflavonoids such as genistein and daidzein have many effects on host-pathogen interactions and displayed resistance against *M. incognita*. During the present study, seedlings were moved to a pot filled with sterilized soil after sampling to prevent further invasion of J2s since it typically takes 3–7 days for plant-parasitic nematodes to migrate, locate, and penetrate the preferred host root *via* root exudate signals (Duan et al., 2011). To further investigate whether J2 entirely invaded the soybean root, roots used for proteomic analysis by iTRAQ were sampled at 7 dpi. The outcomes of the annotation showed that 578, 520, 507, 448, and 294 genes were enriched in the metabolic process, binding, catalytic activity, cell process, and cell part, respectively. In order to suppress a pathogen challenge, plant has a sophisticated immune response strategy and metabolism with interactions among several metabolic pathways. According to the results of protein identification from two treatments, Sneb183 + J2 initiated a difference in types of protein in soybean root, indicated rhizobia, a refined parasite, and triggered soybeans to produce an extensive resistance. The functions of some proteins are involved in flavones and isoflavonoids synthesis pathways are discussed further below (Figure 4).

As the initial enzyme in the phenylpropanoid pathway, PAL catalyzes the precursor of the amino acid L-phenylalanine to cinnamic acid and produces the phenylpropanoid compounds function as preformed or inducible resistance against infection of pathogens (Yadav et al., 2020). In higher plants, PAL catalytic activity affects the efficiency of the isoflavonoid biosynthesis pathway (Jiang et al., 2013). In this study, five PAL were identified to be upregulated in Sneb183 + J2 vs. J2, these proteins provide novel insights into the understanding of the induce resistance in soybean in response to Sneb183. Trans-cinnamate 4-monooxygenase that is also called as cinnamate 4-hydroxylase (C4H) participates in the synthesis of numerous polyphenoid compounds by plants when challenged by pathogens, such as flavones, isoflavonoids, and lignin (Zhu et al., 2020). The accumulation of the enzyme in our study could be related to the accumulation of flavones and isoflavonoids, increasing lignification of the cell wall and then enhance soybean resistance, which is similar to the results in *Ulmus pumila* (Zuo et al., 2019). Moreover, increased expression levels of seven CHS were identified in present study. CHS is a plant-specific polypeptide synthase and the first committed enzyme in regulating biosynthesis and flux control in the isoflavonoid pathway (Schröder, 1997). The involvement of CHS in phytoalexin synthesis has also been reported (Dao et al., 2011; Cho and Lee, 2015). Parasitic nematode fine-tunes the pattern of plant cell death by stimulating host NADPH oxidases to produce reactive oxygen species to limit plant cell death and promote infection (Siddique et al., 2014). It is well-known that POD (peroxidase) is crucial antioxidant enzymes responsible for ROS scavenging (Choudhury et al., 2017), in the present study, of six POD, four were up-regulated and two were suppressed. Similar results were showed in soybean root inoculated by BCA *Bacillus simplex* Sneb545 (Kang et al., 2018). These results suggested that the biosynthesis of isoflavonoids in soybean root was associated with ISR induced by Sneb183.

Throughout this research results, the key genes (*GmPAL*, *GmCHS*, *GmCHR*, and *GmIFS*), the key enzymes in the isoflavonoids pathway (Jiao et al., 2014; Anguraj Vadivel et al., 2019), were upregulated at different stages after treatments, and the synthesis and accumulation of isoflavonoids were induced. But the induction processes and the abundance of isoflavonoids were different among four treatments. *GmIFS*, that is a relatively latter step in the general isoflavonoids biosynthesis, was the first to be upregulated expressed at 3 dpi in Sneb183-treated soybean root and synthesized genistein using the existing precursors in soybean. Until 7–21 dpi, the expression of *GmCHR* and *GmCHS* began to be upregulated in two treatments of with Sneb183 coating, thereby synthesizing the precursor in the phenylpropanoid pathway that has been consumed. Evidence showed that during the initial process, some rhizobia is recognized as possible invaders, then the plant’s immune response is triggered after the active invasion (Zamioudis and Pieterse, 2012), the rhizobia triggering immunity pattern is similar to our findings, once Sneb183 invaded and successfully proliferated in soybean root, it tuned the metabolism and immune response of soybean against pathogens. Consequently, the transcription of *GmCHS* and *GmCHR* were enhanced and biosynthesis of genistein and daidzein increased rapidly as compared to the control and J2.

Both SCN and *S. fredii* Sneb183 infiltrated soybean as soybean parasite, triggering plant resistance and contributing to isoflavonoids accumulation. Interestingly, in the four treatments, we studied the accumulation of two isoflavonoids was initiated by Sneb183-treated were more advanced than that without *S. fredii* Sneb183 coating. That indicated the procedures of inoculation of *S. fredii* Sneb183 and invasion of nematodes to stimulate resistance to soybean are clearly different. Meanwhile, isoflavonoids, especially genistein and daidzein in induced resistance of soybean by *S. fredii* Sneb183 are work together to reduce nematode invasion and inhibit the development of nematodes in soybean root. Some reported genistein and daidzein, more important compounds produced in soybean, were 50 and 40%, respectively (Ren et al., 2001), but in our study, daidzein content is beyond the content of genistein after Sneb183-treated. It is the trait of induced system resistance of *S. fredii* Sneb183 by quickly stimulates the production of isoflavonoids especially daidzein after inoculation. Some researchers recorded that genistein-treated nematode parasites show alterations and deformities in their tegmental architecture (Pal and Tandon, 1998; Kar and Tandon, 2000), thus inducing muscular paralysis around the pharynx and affects stylet puncture and withdraw nutrition (Toner et al., 2009; Tandon and Lyndem, 2010). The content of daidzein increases rapidly in the latter stage of treatment as compared to genistein. While no clear evidence exists to support the essential function of daidzein in nematodes, daidzein was found in our study to significantly kill J2 and inhibit egg hatching (Guo et al., 2017). Evidence reveals that isoflavonoids impede SCN replication by influencing the sex ratio and the number of female eggs (Chin et al., 2018). We, therefore, believe that the rapid accumulation of genistein at 7 days and daidzein at 21 days was jointed to prevent the development of the juvenile of SCN, cyst formation, even subsequent death of nematodes due to unfavorable conditions of nutrients, especially in the treatment of Sneb183. This is consistent with our previous research results that inoculated with Sneb183 fermentation broth; the development of SCN in soybean root was inhibited and declined the number of cysts (Tian et al., 2014). The genistein and daidzein content reached its peak at 35 dpi and inhibited second-generation SCN infection, indicating that the resistance caused by Sneb183 is relatively long-lasting. The susceptible cultivar Liaodou 15 acquired broad resistance, responded immediately against SCN *via* seed coated with *S. fredii* Sneb183. Carter et al. (2018) reported that the SCN resistant genotypes had significantly higher isoflavonoids content than the susceptible genotypes in SCN infested environments.

In summary, to the best of our knowledge, the *S. fredii* Sneb183 induced local and systemic resistance through seed coating with the Sneb183 fermentation. This study is the first report of Sneb183 eliciting isoflavonoids biosynthesis against *H. glycines*. Overall, 456 DEPs were identified relating to metabolic molecular mechanisms that induced the expression of key genes involved in the pathways of isoflavonoid biosynthesis. The content of isoflavonoids was analyzed by HPLC, and the results showed that the *S. fredii* Sneb183 can induce and regulate the synthesis of genistein and daidzein in soybean roots. However, *S. fredii* Sneb183 in inducing ISR against SCN is a complicated and delicate response. The overall results of the present study support *S. fredii* Sneb183 as a potential BCA inducing ISR against *H. glycines*.

## Data Availability Statement

The datasets presented in this study can be found in online repositories. The names of the repository/repositories and accession number(s) can be found in the article/Supplementary Material.

## Author Contributions

YW: method optimization, data analysis, and drafting the manuscript. RY: protein isolation and data analysis. YF: qRT-PCR analysis and data analysis. AS: manuscript preparation and editing. XZ and HF: Sneb183 fermentation and nematodes preparation and seedlings treatment. XL: measurement of genistein and daidzein content by HPLC. LC and YD: overall design of the project and experiments. All authors contributed to the article and approved the submitted version.

### Conflict of Interest

The authors declare that the research was conducted in the absence of any commercial or financial relationships that could be construed as a potential conflict of interest.

## Footnotes

^1^https://www.blast2go.com/

^2^http://wego.genomics.org.cn/

^3^http:/www.kegg.jp/
